# The effect of desulfurization on the postharvest quality and sulfite metabolism in pulp of sulfitated “Feizixiao” Litchi (*Litchi chinensis* Sonn.) fruits

**DOI:** 10.1002/fsn3.1008

**Published:** 2019-04-10

**Authors:** Tao Luo, Shuangshuang Li, Dongmei Han, Xiaomeng Guo, Liang Shuai, Zhenxian Wu

**Affiliations:** ^1^ College of Horticulture, South China Agricultural University/Guangdong Provincial Key Laboratory of Postharvest Science of Fruits and Vegetables/Engineering Research Center for Postharvest Technology of Horticultural Crops in South China Ministry of Education Guangzhou P.R. China; ^2^ Institute of Fruit Tree Research, Guangdong Academy of Agricultural Sciences/Key Laboratory of South Subtropical Fruit Biology and Genetic Resource Utilization Ministry of Agriculture Guangzhou P.R. China; ^3^ Guangdong Litchi Engineering Research Center/Key Laboratory of Biology and Genetic Improvement of Horticultural Crops (South China) of Ministry of Agriculture Guangzhou P.R. China; ^4^ College of Food and Biological Engineering/Institute of Food Science and Engineering Technology Hezhou University Hezhou P.R. China

**Keywords:** desulfurization, *Litchi chinensis* Sonn., postharvest quality, residual sulfite, sulfite metabolism, sulfur fumigation

## Abstract

The residual sulfite caused by sulfur fumigation (SF) is a hazard to health and influenced the export trade of litchi. Desulfurization (DS) is a valid chemical method to reduce the residual sulfite. However, the effect of DS on fumigated litchi has not been studied at physiological and molecular level. This study was aimed to evaluate the effect of DS (SF plus 3% desulfurizer) on the postharvest quality, sulfite residue, and the sulfite metabolism in sulfitated “Feizixiao” litchi during the 4°C storage. Results indicated that the DS promoted the color recovery of sulfitated litchi and achieved an effect similar to SF on controlling rot and browning. DS recovered the water content and respiration rate of sulfitated litchi pericarp. Thus, DS improves commodity properties of sulfitated litchi. Moreover, DS greatly reduced sulfite residue especially in pulp and ensured the edible safety of sulfitated litchi. The activities of sulfite oxidase, sulfite reductase, serine acetyltransferase, and *O*‐acetylserine(thiol) lyase in pulp increased after SF but fell down after DS while the expressions of their encoding genes decreased after SF but then rallied after DS. These results indicated the key role of these enzymes in sulfite metabolism after SF and DS changed the sulfite metabolism at both enzymatic and transcriptional level. It could be concluded that DS used in this study was an effective method for improving the color recovery and ensuring the edible safety of sulfitated litchi by not only chemical reaction but also both of enzymatic and transcriptional regulation.

## 1 INTRODUCTION

Litchi (*Litchi chinensis* Sonn.) fruits were nonclimacteric subtropical fruits which were originated in China and prized on the world market for their flavor, semitranslucent white pulp, and attractive red skin (Yun et al., 2016). However, the short shelf life of litchi fruits under ambient conditions has greatly restricted the development of litchi industry and export trade in China (Jiang, Li, & Li, 2004). Decay and pericarp browning are the main causes of postharvest loss of litchi fruits. Pathogenic microorganisms such as *Peronophythora litchii*, *Geotrichum candidum* Link, *Colletotrichum gloeosporioides* Penz., and *Aspergillus niger* are the mainly biotic stresses leading to postharvest rot of litchi fruits (Wu et al., 2017). Postharvest pericarp browning was mainly caused by the increase in enzymatic activity of laccase, PPO, and POD induced by abiotic and biotic stresses (Fang et al., 2015). Many strategies have been developed to inhibit or delay the postharvest decay and pericarp browning of litchi fruits. Dipping in fungicides (such as imazalil, prochloraz, and benomyl) combined with low temperature storage was effective methods to reduce postharvest loss of litchi fruits. Meanwhile, treatments with oxalic acid (Zheng & Tian, 2006), acetic acid, hydrochloric acid (Jiang, Duan, Joyce, Zhang, & Li, 2004) or other acid solution (Jiang & Fu, 1998), inhibitors of enzymes (NaCl, CaCl_2_, kojic acid) (Reichel et al., 2017), hormones (methyl jasmonate) (Yang et al., 2011), salicylic acid (Kumar, Mishra, Chakraborty, & Kumar, 2011), antioxidants (tea polyphenols) (Chen, Zhang, Shen, Duan, & Jiang, 2004), apple polyphenols (Zhang et al., 2015), tea seed oil (Zhang et al., 2017), ascorbic acid, iso‐ascorbic acid, l‐cysteine, *N*‐acetyl cysteine, kojic acid (Shah, Khan, & Ali, 2017), glutathione (Jiang & Fu, 1998), acidified calcium sulfate (Wang, Chen, Jin, & Gao, 2010), and biocontrol bacterias (*Bacillus subtilis* (Jiang, Zhu, & Li, 2001), *Lactobacillus plantarum* (Martínez‐Castellanos et al., 2011), *Bacillus amyloliquefaciens* (Wu et al., 2017)) have been reported to delay or inhibit the development of litchi pericarp browning. In addition, storage under controlled atmosphere (Jiang & Fu, 1999), pure oxygen (Duan et al., 2004), NO (Duan et al., 2007), or O_3_ (Whangchai, Saengnil, & Uthaibutra, 2006), treatments with chitosan (Zhang & Quantick, 1997), edible coatings, plastic film (Scott, Brown, Chaplin, Wilcox, & Bain, 1982), hydrothermal dipping (Trevor, Nacey, Wiltshire, & O'Brien, 2003), gamma irradiation (Mishra et al., 2012), ultraviolet or ultrasonic have also been reported to be effective to inhibit pericarp browning. However, over the past three decades, SO_2_ fumigation (or dipping with sodium metabisulfite or other SO_2_‐donor) was the most effectively and widely used technology to control the loss the of litchi fruits during long distance transportation and export.

Sulfur dioxide was used widely as preservative and sanitizing agent to prevent spoilage by microorganisms in fruit juices, syrups, wine or vinegar, dehydrated and dried fruits, vegetables, traditional chinese medicine products, table grapes, kiwifruits, blueberries, litchi fruits, and other fresh fruits due to its strong oxidability, low cost, volatileness, operability, and excellent sterilization ability. It was also used as an antioxidant and inhibitor of enzyme‐catalyzed oxidative discoloration and of nonenzymatic browning during preparation, storage, or distribution of many food products (Joslyn & Braverman, 1954). The utilization of SO_2_ had been applied in the marketing of grapes since 1920s and succeeded in commercial preservation of litchi fruits in 1980s (Swarts, 1985). SO_2_ fumigation caused the red color to be bleached to yellow, which was slowly and partially restored to pink. SO_2_ interacted with the membranes, making the rind pliable and leaky to solutes. In addition, SO_2_ directly reacted with anthocyanins and inhibits nonenzymatic formation of colorless quinone–sulfite complexes and enzymatic browning by inactivation of PPO (Jiang et al., 2006). However, due to its reaction with water thereby forming sulfite (the main form of sulfur residue), SO_2_ showed toxicity for organisms (Baillie et al., 2016). The approval from Europe, Australia, and Japan for SO_2_ was likely to be withdrawn due to concerns over sulfur residues in fumigated litchi fruits (Jiang et al., 2006). Therefore, the solution of residual sulfite is the key to break the export barrier of litchi fruits.

In order to restore the color and reduce the sulfur residue, proper desulfurization was often carried out after SO_2_ fumigation. In addition to the chemical desulfurization methods, the enzymatic degradation of sulfite also plays an important role in reducing sulfur residue. SO_2_ gas entered the cell apoplast space and formed sulfite (SO_3_^2−^) with water. Sulfite is a toxic metabolite that can break disulfide bridges, which is termed sulfitolysis; sulfite inhibits numerous enzymes, and it can attach to aldehydes forming hydroxysulfonates, which are metabolic inhibitors (Hänsch et al., 2006). Therefore, its fast removal by oxidation to nontoxic sulfate is a means to protect the cell against excess of sulfite derived from SO_2_. Plants usually relieve the SO_2_ stress in three ways: regulating stomatal conductance to control the amount of SO_2_ entering the cell, oxidizing the SO_3_^2−^ into SO_4_^2−^ by sulfite oxidase (SO) in peroxisome, and then was stored in the vacuoles or converting the SO_3_^2−^ into a thiol or other sulfur‐containing compounds via a reduction pathway (Aghajanzadeh, Hawkesford, & Kok, 2016; Baillie et al., 2016; Chao et al., 2014). Oxidative detoxification produces sulfuric acid (SO_4_^2−^) and radicals (such as H_2_O_2_) which were highly reactive and themselves might cause cellular damage by reacting with essential cell constituents. The SO_4_^2−^ stored in vacuole together with the SO_4_^2− ^absorbed by root system might be transported from the vacuole into the plastids and reduced to SO_3_^2−^ again by adenosine 5′‐phosphosulfate reductase (APR) when the S source was lacked. Also, the SO_3_^2−^ could directly enter the plastids, and this part of SO_3_^2− ^together with the SO_3_^2−^ reduced by APR was further reduced to S^2−^ by sulfite reductase (SiR). The incorporation of reduced sulfur into serine produces cysteine and which might act as a substrate for synthesis of glutathione, methionine, proteins, and other sulfur‐containing cellular constituents. This reduction process involved two enzymatic steps, respectively, conducted by serine acetyltransferase (SAT) and *O*‐acetylserine (thiol) lyase (OAS‐TL). However, these studies focused on the effects of low concentration SO_2_ on sulfur metabolism in plant leaves or roots (Aghajanzadeh et al., 2016; Brychkova, Yarmolinsky, Fluhr, & Sagi, 2012; Randewig et al., 2014), but few reports on the effects of high concentration of SO_2_ on sulfur metabolism in sulfitated fruits especially the edible parts such as pulp. In this study, we performed a desulfurization (DS) treatment to reduce the residual sulfite and investigated its effect on the quality and postharvest physiology of sulfitated litchi fruits. In addition, we explored the key regulation steps of sulfur detoxification in litchi pulp by comparing the difference at enzymatic and transcriptional level of five enzymes related to sulfite degradation between fumigated and desulfurization litchi fruits.

## 2 MATERIALS AND METHODS

### 2.1 Plant materials, postharvest treatments, and samplings

The “Feizixiao” litchi fruits used for the present study were grown following commercial cultivation practices in the same orchard in Conghua district, Guangzhou, China. Commercial mature “Feizixiao” litchi fruits with no damage and no disease were harvested.

Fruits of 15 kg dipped with prochloraz (500 mg/kg) for 2 min were set as controls (CK). Fruits of 30 kilograms were fumigated with 20 g burning sulfur (placed in a foil box) for 25 min in a 3 m^3^ enclosed room. After be cooled at ambient temperature, one half of the sulfitated fruits were set as sulfur fumigated (SF) samples, while the other half of the sulfitated fruits were dipped with 3% desulfurizer solution for 4 min and set as sulfur fumigation plus desulfurization (SF + DS) samples. All of the fruits were packed into 0.025 mm polyethylene bags (30 fruits/bag) and stored at 4°C. After be separated, the pulp and peel were immediately frozen in liquid nitrogen, and kept at −80°C until be used. All samplings at each time point were performed in three biological repeats. The samplings were respectively conducted at 0, 8, 16, 24, 32, 40, and 48 days after storage (DAS).

### 2.2 Chemicals

ZnSO_4_, NaOH, H_2_SO_4_, sodium borate, formaldehyde, pararosaniline, sodium tetrachloromercurate, DTT, KCl, EDTA, EGTA, glycerol, Tris(hydroxymethyl) aminomethane, EDTA‐Na_2_, HCl, K_3_Fe(CN)_6_, Na_2_SO_3_, AMP, cysteine, Triton X‐100, PVP40, HEPES, NADPH, 5,5′‐dithiobis‐(2‐nitrobenzoic acid) (DTNB), acetyl‐CoA, l‐serine, MgCl_2_, Tween 80, PVP, OAS, ninhydrin hydrate, acetonitrile, and methanol were analytically pure and all provided by Sinopharm Chemical Reagent Co., Ltd (Shanghai, China). Deionized water was prepared by distilled water through a Milli‐Q A10 system (Millipore, Milford, MA, USA).

### 2.3 Color measurement

Fruit color was measured by a color analyzer (KONICA MINOLTA CR‐300, Japan). The red to green was expressed as +a* to −a*, yellow to blue was expressed as +b* to −b*, brightness was expressed as L*, and the color index was expressed as CI.

### 2.4 Determination of browning index and rotting rate

Three bags were randomly selected for examining rotting rate. Ten fruits were randomly selected from each bag, and totally thirty fruits were mixed for determination of browning index. All samplings at each time point were performed in three biological repeats. Skin appearance was assessed by measuring the extent of the total browned area on each fruit pericarp of 30 fruit using the following scale: browning grade 0: no obvious browning point on the surface; browning grade 1: small browning points, browning area <25% of total surface area; browning grade 2: obvious browning points (<0.5 cm^2^), browning area: 25%–33% of total surface area; browning grade 3: obvious browning points (0.5–1.0 cm^2^), browning area: 33%–50% of total surface area; browning grade 4: browning points (>1.0 cm^2^), browning area: 50%–75% of total surface area; browning grade 5: browning area >75% total surface area, obvious mildew or flowing liquid. The total browning index of each sample was calculated based on Equation (1):

(1) $$\text{Total}\;\text{Browning}\;\text{index}=\sum \frac{\left(\text{Browning}\;\text{index}\;\text{grade}\times \text{corresponding}\;\text{fruit}\;\text{number}\right)}{\text{Total}\;\text{fruit}\;\text{number}}$$

### 2.5 Peel electroconductibility measurement

The cell membrane permeability of peel was measured according to a method reported by Duan et al. (2004), with some modifications. Three peel disks (diameter 0.5 cm) were punched from each fruit, and peel disks of ten fruits were collected and washed three times by deionized water. Ten peel disks were transferred into a 50 ml tuber containing 25 ml deionized water. 30 min later, the electroconductibility (D1) was measured by a conductivity meter (INESA Instrument DDS‐307, Shanghai, China). Then, the tuber was sealed and subjected to a boiling water bath for 15 min. After be cooled down by ice, the electroconductibility (*D*2) of the solution was measured again. The electroconductibility in another 50 ml tuber containing 25 ml deionized water with no peel disk (set as control sample) (*D*0) was measured. All samplings at each time point were performed in three biological repeats. Relative peel electroconductibility was calculated based on Equation (2):

(2) $$\text{Relative}\;\text{peel}\;\text{electroconductibility}\left(\%\right)=\left(D1-D0\right)/\left(D2-D0\right)\times 100\%$$

### 2.6 Determination of water content in peel

One gram of the peel which was separated from the pulp and cleaned was added into a rapid moisture meter (Satorious MA150). The water content in peel of all samplings at each time point was measured in three biological repeats.

### 2.7 Measurement of respiration rate

Twenty fruits were randomly selected and sealed in a hermetically sealed box for 2 hr at 4°C. The CO_2 _concentration in the gas was determined by a gas chromatography (Shimadzu, GC‐17A) with the conditions as follows: concentric double layer chromatographic column (Molecular sieve 5A combined with Poraoak Q), column temperature: 50°C, carrier gas: helium (He), and thermal conductivity detector (TCD, <150°C). The respiration rate was measured in three biological repeats and calculated based on Equation (3):

(3) $$\text{Respiration}\;\text{rate}\left(\text{mg}\;{\text{kg}}^{-1}\;{\text{h}}^{-1}\right)=\frac{\left(A\times \left(V1-V2\right)\times M\times 273\right)}{\left(H\times W\times 22.4\times \left(273+T\right)\right)}$$

*A*: CO_2 _concentration; *V*1: volume (L) of hermetically sealed box; *V*2: total volume (L) of fruits; *M*: molar mass of CO_2_; *H*: sealed time (hr); *W*: total fruit weight; *T*: storage temperature (°C).

### 2.8 Determination of sulfite residue

The sulfite residue was determined according to a previously reported method (Luo, Li, Guo, Han, & Wu, 2019), with some modifications. 1.0 g peel or 5.0 g pulp was grinded into powder by liquid nitrogen and extracted using 2 ml ZnSO_4_ solution and 5 ml sodium borate solution. The filtrate of the sample was transferred into a volumetric flask (100 ml). Then, 4 ml 0.5 M NaOH solution was added and shaken. After a reaction for 5 min, 4 ml acid solution (H_2_SO_4_:deionized water = 1:71) was added and shaken. After a reaction for 2 min, 20 ml tetrachloromercurate sodium solution was added and shaken, and then the volume was made up to 100 ml by deionized water. After be filtrated, the solution was used for determination of sulfite residue. 5.0 ml tetrachloromercurate sodium solution, 1.0 ml formaldehyde, and 1.0 ml pararosaniline solution were added into 2.0 ml sample solution, mixed fully, and stood still for 10 min. The absorbance of solution at 550 nm was measured by a spectrophotometer. The sulfite residue was calculated based on Equations (4) and (5):

(4) $$\text{Sulphite}\;\text{residue}\;\text{in}\;\text{peel}\;\left(\mu \text{g}\;{\text{g}}^{-1}\;\text{FW}\right)=\frac{\left({\text{OD}}_{550}-0.0002\right)}{\left(0.0336\times 50\right)}$$

(5) $$\text{Sulphite}\;\text{residue}\;\text{in}\;\text{pulp}\;\left(\mu \text{g}\;{\text{g}}^{-1}\;\text{FW}\right)=\frac{\left({\text{OD}}_{550}-0.0002\right)}{\left(0.0336\times 10\right)}$$

### 2.9 Determination of activity of SO

Sample powder (400 mg) grinded by liquid nitrogen was added into 1.6 ml precooled extraction buffer (100 mM Tris‐acetic acid, pH 7.5, containing 10 mM DTT, 10 mM KCl, 1 mM EDTA, 1 mM EGTA, and 10% glycerol), fully mixed. After a centrifugation at 15,700 *g* and 4°C for 20 min, the supernatant was used as crude enzyme extract for assaying activity. Activity of SO was assayed according to a previously reported method (Xia et al., 2012). The absorbance of solution at 420 nm was recorded every minute from 0 to 5 min. The concentration of K_3_Fe(CN)_6_ was calculated by a standard curve. The amount of enzyme required for reduction of 2 μmol K_3_Fe(CN)_6_ every minute was calculated as one activity unit (U).

### 2.10 Determination of activity of APR

Sample powder (400 mg) grinded by liquid nitrogen was added into 12 ml precooled extraction buffer (100 mM Tris‐HCl, pH 7.7, containing 10 mM Na_2_SO_3_, 0.5 mM AMP, 10 mM DTT, 5 mM EDTA‐Na_2_, 10 mM cysteine, 1% Triton X‐100, and 2% PVP40), fully mixed. After a centrifugation at 15,700 *g* and 4°C for 20 min, the supernatant was used as crude enzyme extract for assaying activity. Activity of APR was assayed according to a previously reported method (Scheerer et al., 2010). The absorbance of solution at 420 nm was recorded every minute from 0 to 5 min. The concentration of K_3_Fe(CN)_6_ was calculated by a standard curve. The amount of enzyme required for reduction of 1 μmol K_3_Fe(CN)_6_ every minute was calculated as one activity unit (U).

### 2.11 Determination of activity of SiR

Sample powder (400 mg) grinded by liquid nitrogen was added into 1.6 ml precooled extraction buffer (100 mM Tris‐acetic acid, pH 7.5, containing 10 mM DTT, 10 mM KCl, 1 mM EDTA and 1 mM EGTA and 10% glycerol), fully mixed. After a centrifugation at 15,700 *g* and 4°C for 20 min, the supernatant was used as crude enzyme extract for assaying activity. Activity of SiR was assayed according to a previously reported method (Ostrowski et al., 1989). The absorbance of solution at 340 nm was recorded every minute from 0 to 5 min. Reaction buffer (2.8 ml) mixed with 200 μL deionized water without enzyme extract, and NADPH was setted as control. The concentration of NADPH was calculated by a standard curve. The amount of enzyme required for oxidation of 1 μmol NADPH per minute was calculated as one activity unit (U).

### 2.12 Determination of activity of SAT

Sample powder (400 mg) grinded by liquid nitrogen was added into 1.6 ml precooled extraction buffer (100 mM Tris‐acetic acid, pH 7.5, containing 10 mM DTT, 10 mM KCl, 1 mM EDTA and 1 mM EGTA and 10% glycerol), fully mixed. After a centrifugation at 15,700 *g* and 4°C for 20 min, the supernatant was used as crude enzyme extract for assaying activity of SAT. Activity of SAT was assayed according to a method reported by Randewig et al. (2014). The absorbance of solution at 412 nm was recorded every minute from 0 to 5 min. The concentration of DTNB was calculated by a standard curve. Reaction buffer without l‐serine was setted as control. The amount of enzyme required for reduction of 1 μmol DTNB every minute was calculated as one activity unit (U).

### 2.13 Determination of activity of OAS‐TL

Sample powder (400 mg) together with 20 mg PVP was grinded by liquid nitrogen and then was added into 1.26 ml precooled extraction buffer (100 mM Tris‐HCl, pH 8.0, 100 mM KCl, 20 mM MgCl_2_, 1% Tween 80, and 10 mM DTT), fully mixed. After a centrifugation at 13,400 *g* and 4°C for 10 min, the supernatant was used as crude enzyme extract for assaying activity. Activity of OAS‐TL was assayed according to a method reported by Chronis and Krishnan (2003). The absorbance of solution at 420 nm was recorded every minute from 0 to 5 min. The concentration of K_3_Fe(CN)_6_ was calculated by a standard curve. The amount of enzyme required for reduction of 1 μmol K_3_Fe(CN)_6_ every minute was calculated as one activity unit (U).

### 2.14 RNA isolation and quantitative real time PCR analysis

Total RNA was extracted by using a rapid RNA Extraction Kit 3.0 (Huayueyang Biotechnology CO., LTD, Beijing, China), following the manufacturer's recommendations. Integrity of RNA was electrophoretically verified, and then its concentration, *A*_260_/*A*_280_ and *A*_230_/*A*_260_ absorption were detected by NanoDrop (Agilent 2,100, USA). One μg of total RNA from each sample was used to synthesize the first strand cDNA and eliminate potential DNA contamination using the PrimeScript™ RT reagent Kit with gDNA Eraser (Perfect Real Time; Takara Biomedical Technology Co., Ltd., Beijing, China), following the manufacturer's recommendations. The qRT‐PCR was carried out in a Roche Lightcycler® 480 (Roche Applied Science) using SYBR Green I Master according to the manufacturer's instructions, under the thermal cycle conditions of an initial denaturation at 95°C for 10 min, followed by 40 cycles of 95°C for 10 s, 60°C for 20 s for annealing, and a final step of extension at 72°C for 20 s. The expression levels of the selected genes were calculated by the delta‐delta‐Ct method (Schmittgen & Livak, 2008). The β‐actin gene was used as reference gene for data normalization. Each biological sample was examined in duplicate with three technical replicates. Genes and primers for quantitative reverse transcription‐PCR analysis were listed in Supporting Information Table S1.

### 2.15 Statistical analysis

The variance of data was analyzed using SPSS software package release 16.0 (SPSS Inc. Chicago, IL, USA). Multiple comparisons were performed by one‐way ANOVA based on Duncan's multiple range tests.

## 3 RESULTS AND DISCUSSION

### 3.1 DS accelerated the color recovery of sulfitated fruits and kept the inhibitory effect of SF on browning and decay

**Figure 1:**
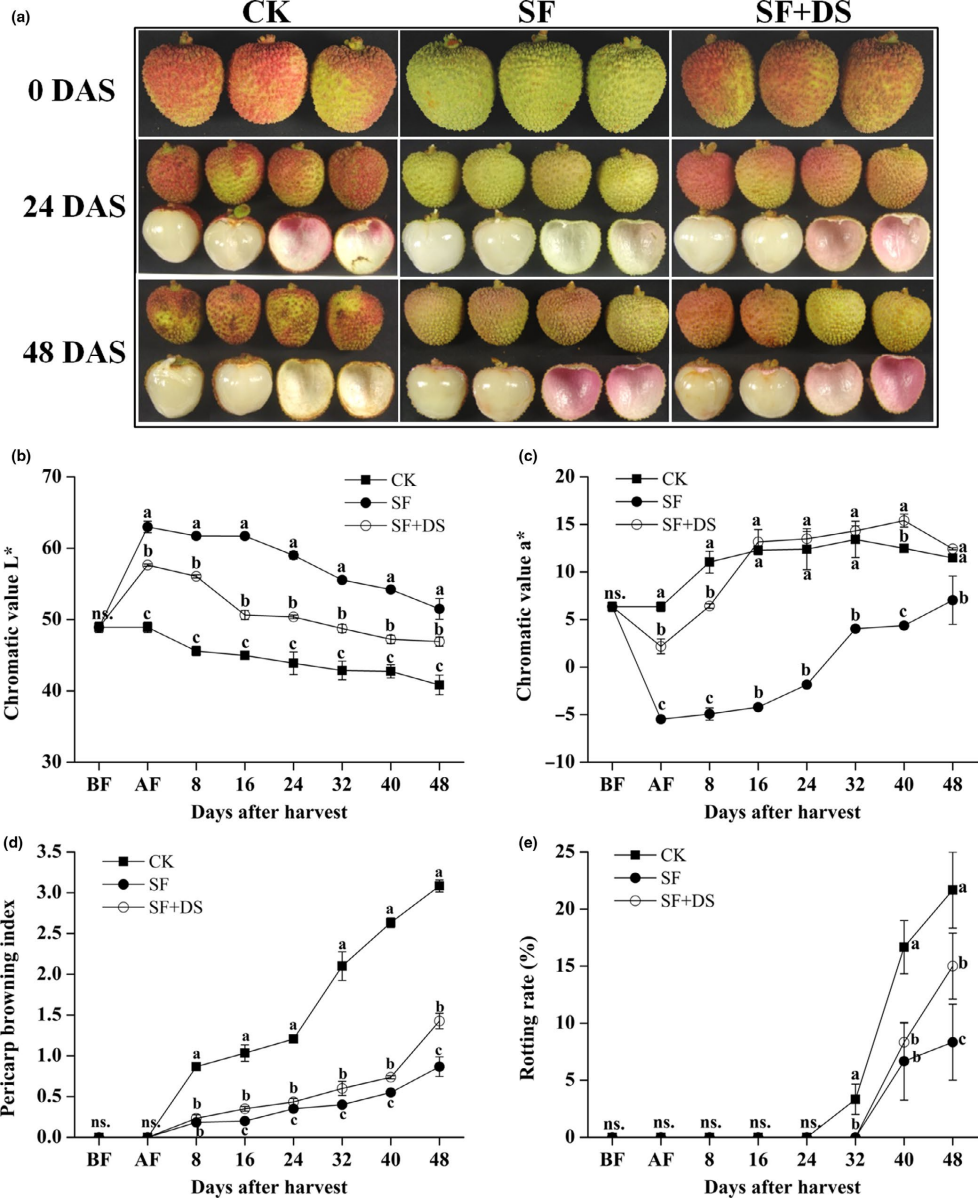
Effect of sulfur fumigation and desulfurization treatment on the appearance (a), chromatic value L* (b), chromatic value a* (c), pericarp browning index (d), and rotting rate (e) of “Feizixiao” litchi (stored at 4°C). Lower case letters after the means designate significance at *p* < 0.05

To investigate the effect of desulfurization on the quality of SF “Feizixiao” litchi, the pigmentation, browning index, and rotting rate were compared among CK, SF, and SF + DS groups. The red pigmentation of “Feizixiao” litchi fruits immediately disappeared and turned to be yellowish green after the sulfur fumigation, but it quickly restored after the desulfurization (0 DAS, AF; Figure 1a). The results of chromatic value indicated a trend that the lightness of “Feizixiao” litchi was SF > SF + DS > CK (Figure 1b), while the a* value was totally CK > SF + DS > SF (Figure 1c). During the low temperature storage, the pericarp browning and decay were effectively inhibited by both of the sulfur fumigation and desulfurization treatment (Figure 1a,d,e). Although the pericarp browning of SF fruits showed a significantly slower increase than that of SF + DS fruits since 16 DAS, the pericarp browning of SF + DS fruits totally showed a far slower increase than that of the CK fruits which showed an obvious browning appearance (pericarp browning index >1) since 16 DAS (Figure 1d; *p* < 0.05). Moreover, the rotting rate of the CK fruits showed an obvious increase since 32 DAS and reached 21.67% at 48 DAS, while that of SF and SF + DS fruits showed obvious increase since 40 DAS and reached only 8.33% and 15% at 48 DAS, respectively (Figure 1e). These results indicated that desulfurization treatment not only effectively restored the pigmentation, but also performed a significantly inhibitory effect on the pericarp browning and decay.

### 3.2 DS totally increased the water content, relative electroconductibility, and respiration rate of sulfitated litchi pericarp

**Figure 2:**
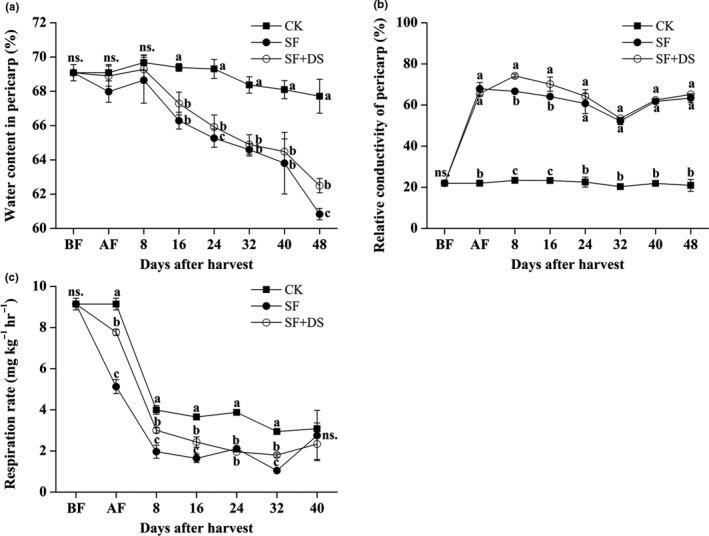
Effect of sulfur fumigation and desulfurization treatment on water content in pericarp (a), relative conductibility of pericarp (b) and respiration rate (c) of “Feizixiao” litchi (stored at 4°C). Lower case letters after the means designate significance at *p* < 0.05

The water content of CK litchi pericarp maintained at a relatively stable level (decreased from 69.08% to 67.71%), while that of the SF and SF + DS litchi pericarp respectively decreased by 7.14% and 6.4% after a 48‐days storage (Figure 2a). The relative electroconductibility of the CK litchi pericarp fluctuated from 20.3% to 23.4%. Both of the SF and SF + DS treatments lead to a higher relative electroconductibility of pericarp which fluctuated from 60.7% to 74.22% during the storage. The relative electroconductibility of the SF + DS litchi pericarp was higher than that of the SF litchi pericarp at 8–24 DAS and showed no significant difference to that of the SF litchi pericarp at 0 DAS (AF) and 24–48 DAS (Figure 2b). The respiration rate of the CK fruits was 9.14 mg kg^−1^ hr^−1^ at 0 DAS (AF) and rapidly decreased to 3.99 mg kg^−1^ hr^−1^ at 8 DAS and then gently decreased to 2.94 mg kg^−1^ hr^−1^ at 48 DAS. The respiration rates of the SF and SF + DS fruits were, respectively, 5.13 and 7.77 mg kg^−1^ hr^−1^ at 0 DAS (AF), and were significantly lower than that of the CK fruits during the whole storage except 48 DAS. The respiration rate of the SF + DS fruits was totally higher than that of the SF fruits except 24 DAS and 48 DAS (Figure 2c). These results indicated that desulfurization might help recovered the water content and respiration rate of SF litchi pericarp, but totally increased the relative electroconductibility.

### 3.3 DS significantly reduced the residual sulfite in the pericarp and pulp of sulfitated litchi fruits

**Figure 3:**
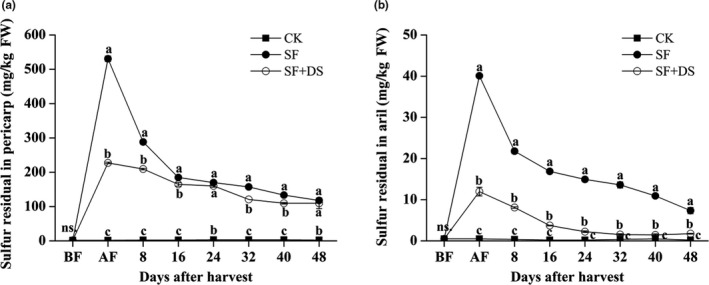
Effect of sulfur fumigation and desulfurization treatment on the sulfur residual in pericarp (a) and pulp (b) of “Feizixiao” litchi (stored at 4°C). Lower case letters after the means designate significance at *p* < 0.05

To examine the effect of desulfurization on the residual SO_2_ of SF “Feizixiao” litchi fruits, the sulfite content in the pericarp and pulp of CK, SF, and SF + DS fruits were investigated during the 48‐day storage. The sulfite content in the CK fruits were almost undetected (the sulfite content in pericarp ≦3.27 mg/kg FW, the sulfite content in pulp ≦0.5 mg/kg FW). The sulfite content in the SF litchi pericarp was 530.8 mg/kg FW at 0 DAS (AF), then decreased to 184.8 mg/kg FW at 16 DAS, and smoothly decreased to 117.86 mg/kg FW at 48 DAS. The sulfite content in the SF + DS litchi pericarp was 227.2 mg/kg FW at 0 DAS (AF) and then smoothly decreased to 109.67 mg/kg FW at 48 DAS. It is noteworthy that the sulfite content in the SF + DS litchi pericarp was totally lower than that in the SF litchi pericarp except 24 and 48 DAS (Figure 3a). The sulfite content in the SF litchi pulp was 40.1 mg/kg FW at 0 DAS (AF), then quickly decreased to 21.78 mg/kg FW at 8 DAS, and decreased to 7.35 mg/kg FW at 48 DAS. The sulfite content in the SF + DS litchi pulp was only 11.96 mg/kg FW at 0 DAS (AF), decreased to 3.7 mg/kg FW at 16 DAS, and was much lower at the later stages (Figure 3b). These results indicated that desulfurization treatment significantly reduced the residual SO_2_ in the pericarp especially in the pulp of SF litchi. The concentration of SO_3_^2−^ in the sulfitated pulp rapidly decreased after desulfurization, while the activity of enzymes for the oxidation and reduction of SO_3_^2−^ decreased simultaneously. This result indicated that the litchi fruit can respond quickly to the SO_2_ stress signal, which is transmitted from pericarp to pulp.

### 3.4 DS significantly reduced the activity of enzymes responsible for detoxification of sulfite in the sulfitated litchi pulp

**Figure 4:**
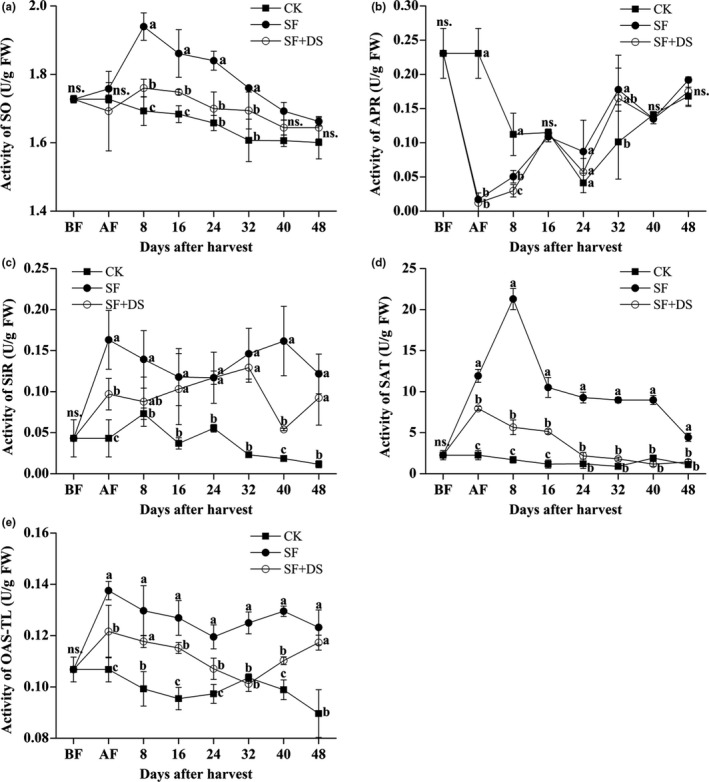
Effect of sulfur fumigation and desulfurization treatment on the activity of five enzymes (SO, APR, SiR, SAT, and OAS‐TL) related to sulfur metabolism in pulp of “Feizixiao” litchi (stored at 4°C). Lower case letters after the means designate significance at *p* < 0.05

The activity of five enzymes related to metabolically detoxification of sulfite in litchi pulp was detected during the storage. The SO activity in the CK litchi pulp showed a smooth decrease during the storage, while that in both of the SF and the SF + DS litchi pulp showed no significance difference to the CK litchi pulp at 0 DAS (AF), but then increased and reached the peak value at 8 DAS and then decreased. In total, the SO activity in the litchi pulp was SF > SF + DS > CK (Figure 4a). The APR activity in the CK litchi pulp fluctuated, decreased, and reached the lowest value at 24 DAS and then increased. The APR activity in both of the SF and the SF + DS litchi pulp was lower than that in the CK pulp by folds at 0 DAS (AF), but then increased and showed higher level than that in the CK pulp only at 32 DAS. The APR activity totally showed no significance difference between the SF pulp and the SF + DS pulp during the storage except 8 DAS (Figure 4b). The activity of SiR, SAT, and OAS‐TL in the litchi pulp was totally SF > SF + DS > CK (Figure 4c–e). The SiR activity in the CK litchi pulp fluctuated and totally decreased during the storage. The SiR activity in the SF litchi pulp was higher than that in the CK pulp by two‐ to threefold during the storage, while the SiR activity in the SF + DS litchi pulp was totally significantly lower than that in the SF pulp but totally significantly higher than that in the CK litchi pulp (Figure 4c). The SAT activity in the CK litchi pulp showed a smooth fluctuation, while that in the SF litchi pulp was higher than that in the CK pulp by more than fourfold at 0 DAS (AF), increased and reached the peak value at 8 DAS and then decreased. The SAT activity in the SF + DS litchi pulp was significantly lower than that in the SF litchi pulp through the whole storage. It is noteworthy that the SAT activity in the SF + DS litchi pulp was higher than that in the CK litchi pulp only at 0 DAS (AF) to 24 DAS (Figure 4d). Also, the OAS‐TL activity in the SF litchi pulp was parallelly higher than that in the CK litchi pulp, while the OAS‐TL activity in the SF + DS litchi pulp was totally lower than that in the SF litchi pulp but totally higher than CK (Figure 4e). The concentration of SO_3_^2−^ in the sulfitated pulp rapidly decreased after desulfurization, while the activity of enzymes for the oxidation and reduction of SO_3_^2−^ decreased simultaneously. This result indicated that the litchi fruits were able to respond quickly to the SO_2_ stress signal, which was transmitted from pericarp to pulp; the upregulated enzyme activity of SO and SAT played an important role in the sulfite metabolism in litchi pulp. Besides the chemical reaction with the sulfite, the DS treatment might also influence the sulfite metabolism by regulation at enzymatic level.

### 3.5 DS significantly recovered the expression of the genes related to detoxification of sulfite in the sulfitated litchi pulp

**Figure 5:**
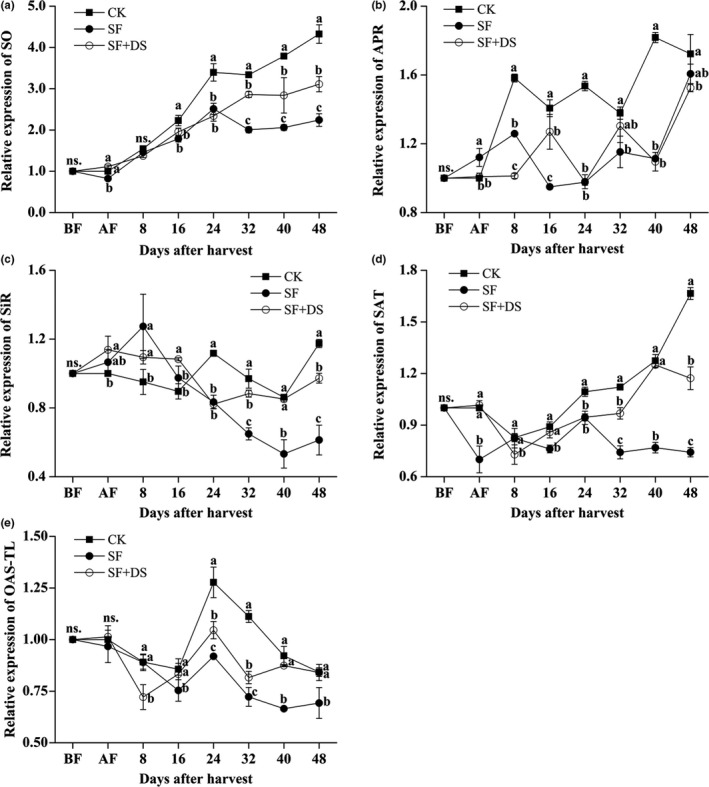
Effect of sulfur fumigation and desulfurization treatment on the expression of five genes (SO, APR, SiR, SAT, and OAS‐TL) related to sulfur metabolism in pulp of “Feizixiao” litchi (stored at 4°C). Lower case letters after the means designate significance at *p* < 0.05

The expression of five genes related to metabolically detoxification of sulfite in the litchi pulp was detected during the storage. Interestingly, the expression of all of the five genes in the SF litchi pulp was totally lower than that in the CK litchi pulp, while the desulfurization treatment significantly recovered the expression of *SO*, *SiR*, *SAT,* and *OAS‐TL* in the SF litchi pulp at the later stages (24–48 DAS), but the expression of all these five genes in the SF + DS litchi pulp was still totally lower than that in the CK litchi pulp (Figure 5). These results indicated an inconsistency between the enzyme activity and expression of sulfite metabolism.

The SO_2_ enters the plant cell through stoma and dissolves in the aqueous phase of the apoplast. It is hydrated to sulfurous acid (SO_3_^2−^) which was cytotoxicity. The sulfate was mainly metabolically detoxified by oxidative reaction into sulfate with increased sulfite oxidase activity or by reductive reaction into S‐metabolites like thiols (Baillie et al., 2016). In this work, our results showed that the activity of SiR, SAT, and OAS‐TL in the litchi pulp increased by folds rapidly after sulfur fumigation. Especially, the activity of SAT increased by more than 10 times after a storage of 8 days and maintained at a relatively stable level later. However, the activity of SO increased slowly after sulfur fumigation. These results were not consistent with the previously reported result that more than 80% of the injected sulfite in arabidopsis and 91% in tomato were oxidized to sulfate which demonstrating the high capacity of the sulfite oxidation mechanisms in plants (Brychkova et al., 2012). Thus, the detoxification mechanisms of sulfite actually vary between model plants and nonmodel plants and between leaves and postharvest fruits. The SO_3_^2−^ in the litchi pulp might be mainly continuously reduced to S^2−^ by increasing the activity of SiR and SAT, and was further converted into cysteine and GSH by SAT/OAS‐TL complex to relieve its cytotoxicity.

## 4 CONCLUSIONS

A comprehensive evaluation of the effect of desulfurization on the storability and the sulfite metabolism in sulfitated and desulfurized fruits is still lacking, while controversial results have been reported in the literature focused on the effects of low concentration SO_2_ on sulfur metabolism in plant leaves or roots. In the present study, our results demonstrate that the optimized desulfurization treatment accelerated the color recovery of sulfitated litchi fruits, achieved an effect similar to sulfur fumigation on controlling rot and fresh‐keeping, and reduced the sulfite residue in the sulfitated litchi so as to ensure its edible safety. The upregulated enzyme activity of SO and SAT, rather than the expression level, plays a central role in the sulfite metabolism of sulfitated litchi pulp. More importantly, DS ensured the edible safety of sulfitated litchi by not only chemical reaction but also both of enzymatic and transcriptional regulation of sulfite metabolism. Conclusions from this study will be helpful to optimize the strategies of sulfur fumigation and desulfurization, and provide a theoretical basis for controlling the sulfur residue in the practices of litchi export.

## CONFLICT OF INTEREST

The authors declare that they have no conflict of interest.

## ETHICAL APPROVAL

The article does not contain any studies with human participants or animals performed by any of the authors.
