# Supplementary material for: The effect of desulfurization on the postharvest quality and sulfite metabolism in pulp of sulfitated “Feizixiao” Litchi (Litchi chinensis Sonn.) fruits
Source: Food Sci Nutr. 2019 Apr 10;7(5):1715–26. doi: 10.1002/fsn3.1008 (PMC6526637; doi:10.1002/fsn3.1008)
Supplement: Supplementary file 1 [file FSN3-7-1715-s001.doc]

**The effect of** **desulfurization on the postharvest quality and** **sulfite metabolism in** **pulp of** **sulfitated ‘Feizixiao’ Litchi** **(*****Litchi chinensis* Sonn.) fruits**

**Running title: SO_2_ metabolism of desulfurized litchi**

Tao Luo^1, #^ **|** Shuangshuang Li^1, #^ **|** Dongmei Han^2^ **|** Xiaomeng Guo^1^ **|** Liang Shuai^4^ **|** Zhenxian Wu^1, 3^, *

^1^College of Horticulture, South China Agricultural University/Guangdong Provincial Key Laboratory of Postharvest Science of Fruits and Vegetables/Engineering Research Center for Postharvest Technology of Horticultural Crops in South China, Ministry of Education, Guangzhou 510642, P.R. China;

^2^Institute of Fruit Tree Research, Guangdong Academy of Agricultural Sciences/Key Laboratory of South Subtropical Fruit Biology and Genetic Resource Utilization, Ministry of Agriculture, Guangzhou 510640, P.R. China;

^3^Guangdong Litchi Engineering Research Center/Key Laboratory of Biology and Genetic Improvement of Horticultural Crops (South China) of Ministry of Agriculture, Guangzhou 510640, P.R. China;

^4^College of Food and Biological Engineering/Institute of Food Science and Engineering Technology, Hezhou University, Hezhou 542899, Guangxi, P.R. China;

^#^These authors contributed equally to this work

*Corresponding authors

**Supplementary Table 1**. Genes and primers for qRT-PCR analysis

| Gene | Forward primer | Reverse primer |
| --- | --- | --- |
| Actin  APR  SO  SiR  SAT  OAS-TL | ACCGTATGAGCAAGGAAATCACTG  GACGGTGAGGAGAAGGAATATG  GAGGAATGTCAGGGCTTCTTTA  GGGAATACCTGACATCCTCAAG  ACCAGCAAAGGTGATAGGATATG  CCAACCCAAAGATCCACTATGA | TCGTCGTACTCACCCTTTGAAATC  GGTACTTGATCGCCTTAGAAGAG  CCATTAGTGGCCTTCTGGTATT  GGCACCCAGTTACCCTTATT  AGAGGGTCTTCCTTCCCTAAA  CACCAGTCCCTATACCAGAAAC |
